# The Impact of the COVID-19 Pandemic on Depressive Symptoms in China: A Longitudinal, Population-Based Study

**DOI:** 10.3389/ijph.2022.1604919

**Published:** 2022-10-04

**Authors:** Yi Zhou, Weicheng Cai, Liyang Xie

**Affiliations:** ^1^ Guanghua School of Management, Peking University, Beijing, China; ^2^ Institute of Behavioral Science, University of Colorado Boulder, Boulder, CO, United States

**Keywords:** COVID-19, China, education, chronic disease, depressive symptoms

## Abstract

**Objectives:** We aimed to examine how COVID-19 incidence is associated with depressive symptoms in China, whether the association is transient, and whether the association differs across groups.

**Methods:** We used a longitudinal sample from 2018 to 2020 waves of the China Family Panel Study. We constructed COVID-19 incidence rates as the number of new cases per 100,000 population in respondents’ resident provinces in the past 7, 14, and 28 days when a respondent was surveyed. We performed linear or logistic regressions to examine the associations, and performed stratified analyses to explore the heterogeneity of the associations.

**Results:** Our sample included 13,655 adults. The 7-day incidence rate was positively associated with the CES-D score (coef. = 2.551, 95% CI: 1.959–3.142), and likelihood of being more depressed (adjusted odds ratio = 6.916, 95% CI: 4.715–10.144). The associations were larger among those with less education, pre-existing depression, or chronic conditions. We did not find any significant association between the 14- or 28-day local incidence rates and depressive symptoms.

**Conclusion:** The impact of COVID-19 incidence on mental health in China’s general population was statistically significant and moderate in magnitude and transient. Disadvantaged groups experienced higher increases in depressive symptoms.

## Introduction

The COVID-19 pandemic and related containment policies have changed almost all aspects of people’s lives. There are increasing concerns worldwide that this pandemic may cause a population mental health crisis [1], resulting from the perceived threat of infection, shortage of medical resources, or other potential losses caused by lockdown, quarantine, and economic difficulties [2–4]. The Chinese government deployed several key policy instruments shortly after the COVID-19 outbreak, including a level 1 emergency declaration, travel bans, and home isolation [5]. According to the information bulletin of the National Health Commission of the People’s Republic of China, 102,314 COVID-19 cases have been identified in mainland China until the end of 2021 [6]. Reliable information from population-level studies is an immediate priority to understand changes in population mental health and for policymakers and medical providers to make appropriate decisions [7].

Recent observational studies from eight countries (China, Spain, Italy, Iran, the United States, Turkey, Nepal, and Denmark) found significant increases in anxiety, depression, post-traumatic stress disorder, psychological distress, and stress during the COVID-19 pandemic [8]. Moreover, mental health deterioration was found to be unequal across groups and possibly worse among vulnerable populations [9–12]. Existing studies that use cross-sectional data found mental health deterioration during the pandemic to be associated with a series of socio-demographic and health-related risk factors such as a younger age, transgender and non-binary gender [13], lower education level, pre-existing physical or psychiatric conditions [8, 14]. Evidence from two studies in the United Kingdom (UK) using national, longitudinal data found that population mental health deteriorated in the first 2 months of the COVID-19 pandemic [15, 16]. However, after the pandemic’s initial shock, the mental health of most UK adults who had experienced mental health deterioration returned to the pre-pandemic level [15]. By tracking geotagged tweets in Australia during the pandemic, a recent study found an opposite pattern for Australian public’s mental health signals [17].

Nevertheless, the evidence from China is limited, as it was derived from cross-sectional data that lacked pre-pandemic information or from convenience samples, or focused on certain populations [4, 18, 19]. Additionally, knowledge of the changing trajectory of mental health and of vulnerable populations in China remain insufficient. Previous studies using either cross-sectional or convenience samples found a high prevalence of mental health disturbance associated with COVID-19 in China among medical and nursing staff [20], high school or college students [21], and the general population [19]. The deterioration of mental health was affecting some groups more intensely, such as pregnant women [22], urban residents [23], and those that use the Internet or smartphone frequently [24]. On one side, when local incidence rate of COVID-19 increased, people would worry about the health consequences associated with infection [25]. Those having chronic conditions would be more worried because the virus was more lethal to them [26]. On the other, a local outbreak also caused economic downturn and social disorders, which could contribute to poor mental health [27], especially among those who had fewer resources [28]. Moreover, people with pre-existing depressive symptoms reacted more sensitively to stressors [29].

Our study is the first to provide evidence using a nationally representative longitudinal sample from China and to explore mental health’s changing trajectories caused by the COVID-19 pandemic for the overall and vulnerable populations.

## Methods

### Study Design and Participants

We used data from the China Family Panel Studies (CFPS), a nationally representative, biennial, longitudinal survey that interviewed about 16,000 households in each wave and carried out six waves since 2010 [30]. To capture individuals’ mental health status associated with the COVID-19 pandemic, we used the most recent wave of data collected from July to December 2020. Interview time was random. Because of the pandemic, only approximately 10.9% of the respondents totaling 25,791 in wave 2020 were interviewed face-to-face, and the rest of the interviews were conducted *via* telephone. The telephone survey date was randomly assigned by a computer. We also used data from the 2018 wave to extract respondents’ pre-pandemic mental health information. We kept the data of adult respondents aged between 18 and 60 as of 2020 considering that 1) children under 16 in each wave completed the questionnaire with the help of their parents; therefore, the information may not be as precise as those collected from respondents older than 16 years and that answered the survey independently, and 2) there might be potential bias from mortality selection or other factors related to retirement. Data on daily provincial COVID-19 cases were from daily reports of provincial centers for disease control and prevention and were manually collected by the authors.

### Measures

The CFPS measures individuals’ mental health condition with the 8-question version of the Center for Epidemiological Studies Depression Scale (CES-D) questionnaire, which has been shown to be valid in the Chinese population [31, 32] and is consistent with the 20-question version of the CES-D. Respondents were asked about the frequency of experiencing eight depressive symptoms in the past week on a four-point scale: 0 (never or less than 1 day), 1 (sometimes or 1–2 days), 2 (often or 3–4 days), or 3 (most of the time or 5–7 days).

The primary outcome of this study was the total score for the CES-D, which was calculated by adding the scores from all eight questions. The possible total scores ranged from 0 to 24 points, with a higher score indicating more severe depressive symptoms. Our secondary outcome was an indicator of having more depressive symptoms (or say “being more depressed”), where the total CES-D score was greater than or equal to 8. We choose the score of 8 points as the cut-off because the average score per question is exactly 1 point in this case.

Our key independent variable was the incidence rate of COVID-19, which was defined as the total number of newly confirmed locally transmitted symptomatic cases per 100,000 population in a given province during a certain period (7-, 14-, or 28-day period) prior to the interview. That is, based on the information of the date when a survey was conducted and the province where a respondent was living, we calculated 7-, 14-, and 28-day incidence rates for each respondent. “Locally transmitted symptomatic cases” referred to local residents who were infected with COVID-19 domestically, distinguished from “imported cases,” which referred to those who just arrived from international travels and were diagnosed with COVID-19 upon arrival or during the quarantine period. We used local cases as our key independent variable and controlled for imported cases in our analysis. The imported cases were not necessarily correlated with local cases, given that overseas travelers were required to be quarantined for at least 14 days, and a negative test was needed for them to be released from quarantine. Importantly, local governments updated the number of new local and imported cases in the media every day. Therefore, the incidence rates can serve as a proxy for respondents’ perceived risk of COVID-19 infection.

Because previous studies had shown that mental health differed across social groups [33], this study also includes several sociodemographic variables, such as age, sex (women vs. men), educational attainment (no school, primary school, junior high school, senior high school, college; or university), marital status (unmarried or cohabitating, currently married, or divorced or widowed), number of children aged below 16 years in the household (0, 1, 2, or ≥3), student status (yes vs. no), employment status (non-entry into the labor market, unemployed, working, or withdrawn from the labor market), hukou status in 2018 (agriculture or urban), and with chronic conditions (yes vs. no) from the 2020 CFPS. The total score for the CES-D questions in 2018 was also calculated.

### Statistical Analyses

Using ordinary least squares (OLS) regression models, we examined the association between the local COVID-19 incidence rate and level of depressive symptoms. We stratified the analysis by 7, 14, and 28 days to identify the dynamics of the temporal relationship between the change in COVID-19 prevalence and people’s mental health reactions. We further explored through stratified analyses whether the association varied by educational attainment (lower than junior high school vs. junior high school and higher), pre-pandemic level of depressive symptoms, or with chronic conditions. These three categories were selected because previous studies have found that these groups were more vulnerable to the health threats of COVID-19 [8, 10]. In addition, we conducted logistic regressions for the binary dependent variable. We adjusted our models for the respondents’ 2018 CES-D scores to control for the pre-pandemic mental health condition and a series of sociodemographic characteristics listed above. We also controlled for the incidence rate of imported cases in the same window within the same province. To alleviate the impact of within-province correlation of the error, we cluster standard errors at the province level [34, 35].

We conducted a series of sensitivity analyses to determine whether the observed association between the COVID-19 incidence rate and depressive symptoms was robust to different settings. First, our results might be biased if there were time-invariant, omitted variables. We replaced the dependent variable with the difference between the 2018 and 2020 CES-D scores and then reran the regression analyses. Moreover, approximately 62.6% of the respondents in the 2018 CFPS were followed in the 2020 CFPS, and we compared the characteristics of the respondents who participated in both surveys and those who only participated in the 2018 CFPS to ensure that there was no severe selection bias in our longitudinal sample.

To explore mental health’s changing trajectory, we constructed 7-, 14-, and 28-day provincial level incidence rates of COVID-19 for each respondent by combining the information of specific telephone interview dates in mid-2020 and the provincial daily COVID-19 cases from the Chinese Center for Disease Control and Prevention. If the impact on mental health is relatively transient, the coefficient of 28-day incidence rate should be much weaker than those of 7-day and 14-day incidence rates. In contrast, if the impact lasts long, the coefficients should be close to each other.

Statistical analyses were performed in Stata, version 17. The CFPS data, which are collected and managed by the Institute of Social Science Survey at Peking University, are publicly available on http://www.isss.pku.edu.cn/cfps/ for academic purpose.

### Patient and Public Involvement

No patients or members of the public were directly involved in the design, conduct, or reporting of this study.

## Results

After excluding those with missing values in either outcome or independent variables, our analytic sample consisted of 13,655 respondents (Table 1). The mean age of our population was 40.81 years, and 48.81% were men. Regarding educational attainment, approximately 37.62% of the sample graduated from senior high school or above. Nearly 79% of the respondents were married. About two-thirds of the respondents reported at least one child aged below 16 years in their households. Regarding employment status, 80.51% of the respondents reported being employed. Approximately 74.78% of the respondents held a rural hukou in 2018. With respect to mental health conditions, the average CES-D score increased slightly, from 5.47 in 2018 to 5.54 in 2020. The average 7-, 14-, and 28-day incidence rates in our sample were 0.0032 per 100,000 (SD = 0.019), 0.0058 per 100,000 (SD = 0.030), and 0.010 per 100,000 (SD = 0.047), respectively.

**TABLE 1:** Social and health characteristics of participants (China Family Panel Study, China. 2018 & 2020).

Characteristics	All sample (*N* = 13,655)
Demographics	
Mean Age, Years (SD)	40.81 (11.97)
Gender	
Men	6,665 (48.81%)
Women	6,990 (51.19%)
Education Attainment	
No School	1,666 (12.20%)
Primary School	2,319 (16.98%)
Junior High School	4,533 (33.20%)
Senior High School	2,647 (19.38%)
College	1,290 (9.45%)
University	1,200 (8.79%)
Marriage Status	
Unmarried/Cohabitation	2,276 (16.67%)
Currently Married	10,765 (78.84%)
Divorce/Widow	614 (4.50%)
Number of Children below 16 in the Household	
0	4,623 (33.86%)
1	4,037 (29.56%)
2	2,818 (20.64%)
≥3	2,177 (15.94%)
Whether Registering into the Full-Time School	
Yes	12,870 (94.25%)
No	785 (5.75%)
Employment Status	
Non-Entry into the Labor Market	754 (5.52%)
Unemployment	196 (1.44%)
In Work	10,993 (80.51%)
Withdrawal from the Labor Market	1,712 (12.54%)
Hukou Status 2018	
Agriculture	10,211 (74.78%)
Urban	3,444 (25.22%)
Having Chronic Conditions	
Yes	1,584 (11.60%)
No	12,071 (88.40%)
Depression Measure	
Mean Score of CES-D 2020 (SD)	5.54 (3.96)
Mean Score of CES-D 2018 (SD)	5.47 (3.74)
Indicator of Being More Depressed (CES-D≥8) in 2020	3,926 (28.75%)
Indicator of Being More Depressed (CES-D≥8) in 2018	3,627 (26.56%)
COVID-19 Exposure	
7-day Local Incidence Rate of COVID-19 (SD)	0.0032 (0.019)
14-day Local Incidence Rate of COVID-19 (SD)	0.0058 (0.030)
28-day Local Incidence Rate of COVID-19 (SD)	0.010 (0.047)
Other COVID-19 Relevance	
7-day Imported Incidence Rate of COVID-19 (SD)	0.0066 (0.023)
14-day Imported Incidence Rate of COVID-19 (SD)	0.012 (0.039)
28-day Imported Incidence Rate of COVID-19 (SD)	0.023 (0.064)

Note: SD, Standard Deviation. Data are n (%) unless specified. Local and imported incidence rates of COVID-19 were constructed by taking the number of emerging local cases per 100,000 population in respondent’s resident province within the specific window.

**FIGURE 1:**
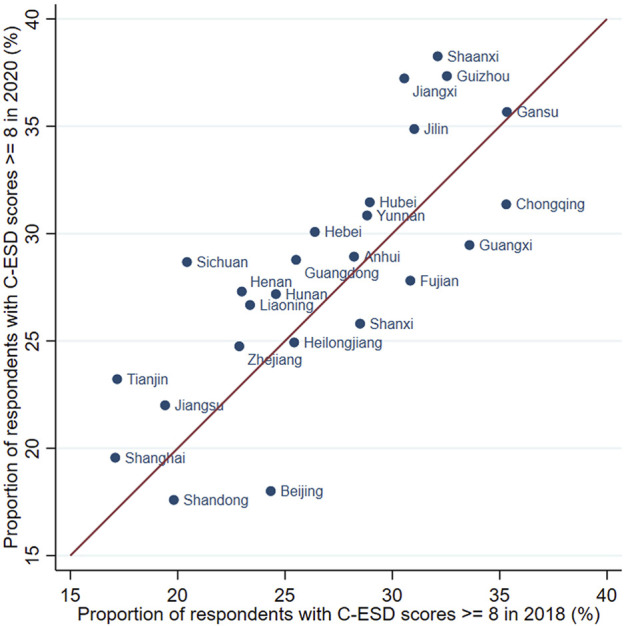
The proportion of more-depressed respondents before and during the pandemic, by province (China Family Panel Study, China. 2018 & 2020) Source: China Family Panel Survey (2018 and 2020 waves).

Figure 1 is a scatter plot showing the province-level proportion of respondents with more depressive symptoms (CES-D>8) before and during the COVID-19 pandemic. We could see that the proportion of being more depressed differed significantly across provinces. The dots are located around the line of 45-degree angle. First, it suggests that the differences in the prevalence of depression across provinces were relatively stable across years. Second, the prevalence of depression did not have a significant change in most provinces after the pandemic.

In Table 2, We show the results of the associations between depression and the 7-, 14-, and 28-day COVID-19 incidence rates from adjusted OLS and logistic regressions. We found that the 7-day incidence rate was positively associated with the CES-D score for depressive symptoms (Table 2, Panel A). The coefficient was 2.55 (95% CI: 1.96–3.14), meaning that the CES-D score would increase by 0.047 points if the 7-day incidence rate increased by one standard deviation. The relationship between the 14-day incidence rate and the CES-D score was still positive but shrunk to 1.72 (95% CI: 0.94–2.50). Meanwhile, the coefficient of the 28-day incidence rate and the CES-D score became even smaller, but not precisely estimated. In general, the impact of COVID-19 incidence was strongest in the first week and faded gradually later. Columns (4)–(6) report the adjusted odds ratios from the logistic regressions. The results were consistent with those of the linear regressions (7-day incidence rate, AOR = 6.916, 95% CI: 4.715–10.144; 14-day incidence rate, AOR = 6.302, 95% CI: 3.333–11.916; 28-day incidence rate, AOR = 2.823, 95% CI: 1.127–7.074). We found that the likelihood of being more depressed increased with the COVID-19 incidence rates, but the size also became smaller over time. The sensitivity analysis using 0–7, 8–14, 15–21, or 22–28-day local incidence rate as independent variables showed consistent patterns (see the results in Supplementary Appendix Tables SA1, SA2).

**TABLE 2:** Associations of COVID-19 incidence rate and depression with OLS model and Logit model (China Family Panel Study, China. 2018 & 2020).

(1)		(2)	(3)	(4)	(5)	(6)
Model		OLS (Coef.)			Logit (AOR)	
Dependent variables		Score of CES-D 2020			Indicator of Being More Depressed in 2020 (CES-D ≥ 8)	
7-day local incidence rate of COVID-19	2.551***			6.916***		
	(1.959–3.142)			(4.715–10.14)		
14-day local incidence rate of COVID-19	0.856			5.691***		
	(−0.942–2.655)			(1.556–20.81)		
28-day local incidence rate of COVID-19		1.720***			6.302***	
		(0.943–2.498)			(3.333–11.92)	
7-day imported incidence rate of COVID-19		−0.0180			2.140*	
		(−1.198–1.162)			(0.884–5.178)	
14-day imported incidence rate of COVID-19			0.705**			2.823**
			(0.114–1.297)			(1.127–7.074)
28-day imported incidence rate of COVID-19			−0.420			1.153
			(−1.471–0.631)			(0.382–3.482)
CES-D scores 2018	0.461***	0.461***	0.461***	1.275***	1.275***	1.275***
	(0.431–0.491)	(0.431–0.491)	(0.431–0.491)	(1.248–1.302)	(1.248–1.302)	(1.248–1.302)
Male	−0.165**	−0.165**	−0.166**	0.898***	0.898***	0.898***
	(−0.299 to −0.0322)	(−0.298 to −0.0321)	(−0.299 to −0.0325)	(0.838–0.962)	(0.839–0.962)	(0.838–0.962)
Age	−0.0241	−0.0243	−0.0244	0.984	0.984	0.984
	(−0.0760–0.0277)	(−0.0761–0.0275)	(−0.0761–0.0273)	(0.949–1.020)	(0.949–1.019)	(0.949–1.019)
Age square	8.98e-05	9.26e-05	9.46e-05	1.000	1.000	1.000
	(−0.000531–0.000711)	(−0.000528–0.000713)	(−0.000525–0.000715)	(1.000–1.001)	(1.000–1.001)	(1.000–1.001)
Education attainment (ref: no school)						
Primary school	−0.0454	−0.0457	−0.0443	1.068	1.067	1.067
	(−0.326–0.235)	(−0.326–0.235)	(−0.325–0.236)	(0.910–1.252)	(0.910–1.251)	(0.910–1.252)
Junior high school	−0.501***	−0.501***	−0.500***	0.869*	0.869*	0.870*
	(−0.768 to −0.234)	(−0.768 to −0.234)	(−0.767 to −0.233)	(0.745–1.015)	(0.744–1.014)	(0.745–1.016)
Senior high school	−0.666***	−0.667***	−0.666***	0.726***	0.725***	0.725***
	(−0.949 to −0.383)	(−0.950 to −0.383)	(−0.949 to −0.383)	(0.611–0.862)	(0.611–0.861)	(0.611–0.861)
College	−0.758***	−0.759***	−0.759***	0.697***	0.696***	0.696***
	(−1.113 to −0.404)	(−1.115 to −0.404)	(−1.113 to −0.404)	(0.557–0.872)	(0.556–0.873)	(0.555–0.872)
University	−0.997***	−0.998***	−0.997***	0.565***	0.564***	0.565***
	(−1.312 to −0.681)	(−1.315 to −0.681)	(−1.313 to −0.680)	(0.448–0.712)	(0.447–0.713)	(0.448–0.713)
Marriage status (ref: unmarried/cohabitation)						
Currently married	−0.224	−0.223	−0.224	0.814	0.814	0.813
	(−0.535–0.0864)	(−0.534–0.0875)	(−0.535–0.0869)	(0.622–1.064)	(0.622–1.065)	(0.622–1.064)
Divorce/Widow	0.785***	0.788***	0.789***	1.403*	1.404*	1.406*
	(0.258–1.313)	(0.260–1.316)	(0.262–1.317)	(0.987–1.992)	(0.988–1.995)	(0.990–1.996)
Number of children below 16 in the household (ref: 0)						
1	0.0858	0.0855	0.0839	1.041	1.041	1.039
	(−0.0553–0.227)	(−0.0558–0.227)	(−0.0550–0.223)	(0.952–1.138)	(0.952–1.139)	(0.952–1.135)
2	−0.0168	−0.0170	−0.0170	0.995	0.995	0.994
	(−0.262–0.228)	(−0.262–0.228)	(−0.262–0.228)	(0.859–1.153)	(0.859–1.151)	(0.859–1.150)
≥3	−4.91e-05	−0.000522	−0.00142	1.062	1.062	1.060
	(−0.220–0.220)	(−0.221–0.220)	(−0.221–0.218)	(0.950–1.188)	(0.949–1.188)	(0.948–1.186)
Not in full-time school	0.816	0.812	0.799	1.209	1.209	1.207
	(−0.193–1.824)	(−0.199–1.823)	(−0.221–1.819)	(0.536–2.727)	(0.535–2.732)	(0.531–2.743)
Employment status (ref: unemployment)						
In work	−0.606***	−0.607***	−0.604***	0.722**	0.722**	0.724**
	(−1.016 to −0.197)	(−1.016 to −0.197)	(−1.012 to −0.197)	(0.536–0.974)	(0.535–0.975)	(0.536–0.977)
Withdrawal from the labor market	−0.577**	−0.578**	−0.576**	0.706**	0.705**	0.706**
	(−1.081 to −0.0738)	(−1.083 to −0.0729)	(−1.079 to −0.0736)	(0.507–0.983)	(0.504–0.985)	(0.506–0.985)
Non-entry into labor markets	−0.984*	−0.986*	−0.998*	0.398*	0.398*	0.398*
	(−2.029–0.0621)	(−2.034–0.0621)	(−2.056–0.0605)	(0.145–1.096)	(0.144–1.098)	(0.144–1.103)
Rural Hukou	0.0761	0.0769	0.0758	1.005	1.006	1.006
	(−0.135–0.287)	(−0.133–0.287)	(−0.136–0.288)	(0.856–1.180)	(0.858–1.180)	(0.857–1.180)
Having chronic conditions	1.177***	1.175***	1.174***	1.719***	1.718***	1.714***
	(0.877–1.476)	(0.875–1.475)	(0.874–1.475)	(1.478–2.001)	(1.475–2.000)	(1.471–1.998)
Constant	3.226***	3.227***	3.169***	0.132***	0.131***	0.115***
	(1.768–4.683)	(1.767–4.688)	(1.714–4.624)	(0.0510–0.342)	(0.0503–0.339)	(0.0449–0.293)
Province FE	Yes	Yes	Yes	Yes	Yes	Yes
Month FE	Yes	Yes	Yes	Yes	Yes	Yes
Observations	13,655	13,655	13,655	13,655	13,655	13,655
R-squared	0.255	0.255	0.255	0.155	0.155	0.155

Note: OLS, Ordinary Least Squares; AOR, Adjusted Odds Ratio. Standard errors are reported in parentheses. *<0.1, **<0.05, ***<0.01. Local incidence rate of COVID-19 was constructed by taking the number of emerging local cases per 100,000 people in respondent’s resident province within the specific window. Standard errors were clustered at the province level.

**FIGURE 2:**
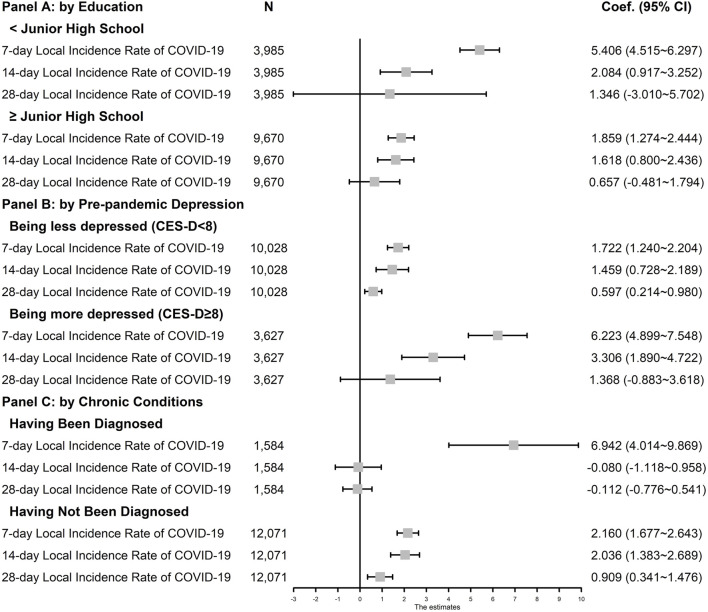
Effects of COVID-19 Exposure on The Likelihood of Being More Depressed by Education, Pre-pandemic Depression, and Chronic Conditions (China Family Panel Study, China. 2018 & 2020) Source: China Family Panel Study (2018 and 2020 waves) and National Health Commission of the People’s Republic of China. Note: Coef., Coefficient. 95% CI = 95% Confidence Interval. 95% CI are reported in parentheses. Local incidence rate of COVID-19 was constructed by taking the number of emerging local cases per 100,000 population in respondent’s resident province within the specific window. Ordinary Least Squares models controlled for: the CES-D score in 2018 wave, age, the square of age, gender, education attainment, marriage status, number of children under 16 years old in the household, whether registering into the full-time school, employment status, hukou status in 2018, whether having chronic disease diagnosed by doctors, the incidence rate of imported cases in the same window period, the province fixed effects, and the month fixed effects. Standard errors were clustered at the province level.

Figure 2 presents the adjusted coefficients from stratified analyses. We found significant increases in depressive symptom scores associated with the 7-day (coef. = 5.406, 95% CI: 4.515–6.297) and 14-day (coef. = 2.084, 95% CI: 0.917–3.252) COVID-19 incidence rates among those with education lower than junior high school. The coefficients were smaller for respondents who had finished junior high school (7-day incidence rate, coef. = 1.859, 95% CI: 1.274–2.444; 14-day incidence rate, coef. = 1.618, 95% CI: 0.800–2.436). Among those who were more depressed in 2018, we found significant increases in depressive symptoms with the 7-day (coef. = 6.223, 95% CI: 4.899–7.548) and 14-day (coef. = 3.306, 95% CI: 1.890–4.722) COVID-19 incident rates. For those who were less depressed, the impact of COVID-19 incidence was much smaller (7-day incidence rate, coef. = 1.722, 95% CI: 1.240–2.204; 14-day incidence rate, coef. = 1.459, 95% CI: 0.728–2.189). Our stratified analyses also revealed a much larger impact of the 7-day COVID-19 incidence rate among those with any chronic condition (coef. = 6.942, 95% CI: 4.014–9.869), compared with those not having any chronic condition (coef. = 2.160, 95% CI: 1.677–2.643). We report the full results of stratified analyses in theSupplementary Appendix Tables SA3–SA5 for conciseness.

The results of the sensitivity analyses are presented in the appendix. Our findings still hold when the CES-D score was changed from that in 2018 to that in 2020 as the dependent variable (Supplementary Appendix Table SA6). Those who were sampled but did not respond to the 2020 wave and those who responded to the 2020 wave were similar in age, gender, hukou status, CES-D score, and the chance of having chronic conditions as of 2018, although those who responded had a slightly higher education (Supplementary Appendix Table SA7). We repeated the statistical analyses with the sample of 3,450 respondents who were surveyed *via* telephone both in 2018 and 2020, and also with a weighted subsample which could keep national representativeness better. We obtained consistent results (Supplementary Appendix Tables SA8, SA9). To explore the potential non-linearity of the impact, we classified the non-zero incidence rates into two groups, high-incidence and low-incidence, and then constructed two dummy variables correspondingly. The results in Supplementary Appendix Table SA10 suggest that, the effects are only statistically significant in the high-incidence group. We also employed random-effects model for robustness check and obtained similar conclusions (Supplementary Appendix Table SA11).

## Discussion

Using nationally representative longitudinal data collected in 2018 and July to December 2020, this study is among the first to investigate the impact of COVID-19 incidence on mental health in China’s general population. We found that the association between the COVID-19 incidence rate and increased depressive symptoms was statistically significant but moderate in magnitude. Moreover, mental health deterioration only lasted for a couple of weeks and reverted to normality within 28 days, suggesting that the negative psychological reactions to COVID-19 incidence was transient. Because the order of telephone interview in CFPS 2020 was randomly assigned by computer, we could consider the timing of interview and local COVID-19 incidence as exogenous [36]. In other words, our research design was like a quasi-experiment and the results were less likely to include biases induced by reverse causalities or omitted variables.

Our results suggest that even in China, where the COVID-19 prevalence was low, mental distress would still be greater among those with a lower education level, pre-existing, more depressive symptoms, or chronic conditions. Different reasons may explain why individuals of these subgroups experienced worse mental distress during a local outbreak of COVID-19. First, people with a lower education level might be more likely to suffer from economic hardships during COVID lockdowns, such as workload decrease and income loss [37]. Furthermore, the risk of COVID-19 infection could impose a heavier emotional burden on people with pre-existing depression because they are more likely to perceive their symptoms to be COVID-19-related [10]. People with chronic conditions were more vulnerable to the COVID-19 pandemic because COVID-related mortality was higher for them and because the local outbreak of COVID-19 delayed their access to medical care [38].

The longitudinal and nationally representative dataset allowed us to track depressive symptoms both before and during the COVID-19 pandemic for the same person and apply our findings to the general Chinese population. Unlike previous studies that only compared the prevalence of depression at various stages of the COVID-19 pandemic, we constructed local incidence rates for each respondent using temporal and regional variations in COVID-19, which captured the perceived risk of infection more precisely. In addition, 87.5% of the 2020 CFPS respondents were surveyed on the telephone in a random order assigned by a computer, making our research design close to a natural experiment. Moreover, our design that controlled for pre-existing depressive symptoms excluded the omitted individual characteristics that might drive the positive association.

We acknowledge that this study has some limitations. First, because of the COVID-19 pandemic, more respondents were surveyed by telephone in the 2020 CFPS than in the 2018 CFPS. The proportion of adult respondents who were surveyed *via* telephone was only 21.8% in the 2018 CFPS, but as high as 89.1% in the 2020 CFPS. People might have different responses to depression-related questions in the face-to-face than in the telephone survey. The statistical analyses with the sample of the respondents who were surveyed *via* telephone both in 2018 and 2020 generated consistent findings (Supplementary Appendix Table SA8). We also used a weighted subsample which could keep national representativeness and obtained similar conclusions (Supplementary Appendix Table SA9). Better Second, the follow-up rate of the 2020 CFPS was 54.8%—lower than all previous waves, which were 77.93% for the 2018 CFPS and 80.99% for the 2016 CFPS. We might have underestimated the prevalence of depression during the pandemic because respondents with more depressive symptoms possibly dropped out. After comparing the characteristics of those who had responded to the 2020 CFPS and those who had been sampled but did not respond, we found that these two groups were not significantly different in most demographic variables. Those who had been followed in the 2020 CFPS had a slightly higher education level (Supplementary Appendix Table SA7). Because individuals who had dropped out of the survey had a lower education level and should have responded to COVID-19 incidence more sensitively according to our subgroup analysis, a lower follow-up rate would cause an underestimation of the impact rather than an overestimation.

This study was conducted in a particular setting where the COVID-19 incidence rate had been controlled at a low level, which might limit the extrapolation of our findings to the rest of the world. However, it has policy implications for psychological interventions in the future stages of the COVID pandemic. Our findings suggest that, even in scenarios where the incidence rate is very small, vulnerable groups might be still struggling emotionally with the COVID-19-driven issues. Meanwhile, mental health inequality would continue to grow during the pandemic because people with pre-existing mental disorders are less tolerant of the uncertainty caused by the COVID pandemic. Therefore, governments and health professionals need to help citizens cope with the short-term negative emotions associated with the risk of COVID-19 outbreak and especially provide support to disadvantaged groups.
