# Supplementary material for: The Impact of the COVID-19 Pandemic on Depressive Symptoms in China: A Longitudinal, Population-Based Study
Source: Int J Public Health. 2022 Oct 4;67:1604919. doi: 10.3389/ijph.2022.1604919 (PMC9576838; doi:10.3389/ijph.2022.1604919)
Supplement: Supplementary file 1 [file DataSheet1.pdf]

Appendix

"The Impact of the COVID-19 Pandemic on Depressive Symptoms in China: A Longitudinal, Population-based Study"

Table A1. Splitting the Local Incidence Rate into Four Weeks in OLS models (China Family Panel Study, China. 2018 & 2020)

	(1)	(2)	(3)	(4)
	Score of CES-D 2020 as Dependent Variable			
	Coef. (95% CI)	Coef. (95% CI)	Coef. (95% CI)	Coef. (95% CI)
0~7-day Local Incidence Rate of COVID-19	2.551<br>(1.959~3.142)			
8~14-day Local Incidence Rate of COVID-19		1.753<br>(0.318~3.188)		
15~21-day Local Incidence Rate of COVID-19			-1.195<br>(-3.226~0.836)	
22~28-day Local Incidence Rate of COVID-19				0.526<br>(-0.485~1.537)

Note: OLS = Ordinary Least Squares. Coef. = Coefficient. 95% CI = 95% Confidence Interval. 95% CI are reported in parentheses. Local incidence rate of COVID-19 was constructed by taking the number of emerging local cases per 100,000 population in respondent's resident province within the specific window. Models controlled for: the CES-D score in 2018 wave, age, the square of age, gender, education attainment, marriage status, number of children under 16 years old in the household, whether registering into the full-time school, employment status, hukou status in 2018, whether having chronic disease diagnosed by doctors, the incidence rate of imported cases in the same window period, the province fixed effects, and the month fixed effects. Standard errors were clustered at the province level.

Table A2. Splitting the Local Incidence Rate into Four Weeks in Logit Models (China Family Panel Study, China. 2018 & 2020)

	(1)	(2)	(3)	(4)
	Indicator of Being More Depressed (CES-D>=8) in 2020 as Dependent Variable			
	AOR (95% CI)	AOR (95% CI)	AOR (95% CI)	AOR (95% CI)
0~7-day Local Incidence Rate of COVID-19	6.916			
	(4.715~10.144)			
8~14-day Local Incidence Rate of COVID-19		16.107		
		(4.254~60.986)		
15~21-day Local Incidence Rate of COVID-19			1.038	
			(0.556~1.936)	
22~28-day Local Incidence Rate of COVID-19				2.110
				(1.150~3.872)

Note: AOR = Adjusted Odds Ratio. 95% CI = 95% Confidence Interval. 95% CI are reported in parentheses. Local incidence rate of COVID-19 was constructed by taking the number of emerging local cases per 100,000 population in respondent's resident province within the specific window. Models controlled for: the CES-D score in 2018 wave, age, the square of age, gender, education attainment, marriage status, number of children under 16 years old in the household, whether registering into the full-time school, employment status, hukou status in 2018, whether having chronic disease diagnosed by doctors, the incidence rate of imported cases in the same window period, the province fixed effects, and the month fixed effects. Standard errors were clustered at the province level.

Table A3. Associations of COVID-19 Incidence Rate and Depression with OLS Model, by education (China Family Panel Study, China. 2018 & 2020)

	(1)	(2)	(3)	(4)	(5)	(6)
Model	OLS (Coef.)					
Dependent variable	*Score of CES-D 2020*					
Subsample	< Junior high school			⩾ Junior high school		
7-day local incidence rate of COVID-19	5.406***			1.859***		
	(4.515 - 6.297)			(1.274 - 2.444)		
14-day local incidence rate of COVID-19		2.084***			1.618***	
		(0.917 - 3.252)			(0.800 - 2.436)	
28-day local incidence rate of COVID-19			1.346			0.657
			(-3.010 - 5.702)			(-0.481 - 1.794)
7-day imported incidence rate of COVID-19	1.882			0.577		
	(-5.895 - 9.659)			(-1.033 - 2.186)		
14-day imported incidence rate of COVID-19		-3.855			0.429	
		(-10.08 - 2.373)			(-0.445 - 1.304)	
28-day imported incidence rate of COVID-19			-5.344			0.277
			(-11.84 - 1.148)			(-0.341 - 0.895)
CES-D scores 2018	0.449***	0.450***	0.450***	0.464***	0.464***	0.464***
	(0.407 - 0.491)	(0.408 - 0.491)	(0.408 - 0.491)	(0.438 - 0.491)	(0.438 - 0.491)	(0.438 - 0.491)
Male	-0.106	-0.106	-0.108	-0.212***	-0.212***	-0.212***
	(-0.433 - 0.220)	(-0.432 - 0.221)	(-0.434 - 0.218)	(-0.316 - -0.108)	(-0.316 - -0.108)	(-0.317 - -0.108)
Age	-0.0954*	-0.0965*	-0.0980*	-0.00770	-0.00775	-0.00800
	(-0.209 - 0.0185)	(-0.210 - 0.0170)	(-0.212 - 0.0157)	(-0.0704 - 0.0550)	(-0.0705 - 0.0550)	(-0.0706 - 0.0546)
Age square	0.000948	0.000960	0.000981	-0.000115	-0.000114	-0.000110
	(-0.000370 -	(-0.000353 -	(-0.000336 -	(-0.000868 -	(-0.000867 -	(-0.000862 -
	0.00227)	0.00227)	0.00230)	0.000638)	0.000639)	0.000642)
Education attainment						
(ref: no school)						
Primary school	-0.0571	-0.0540	-0.0499	-	-	-
	(-0.350 - 0.236)	(-0.348 - 0.240)	(-0.343 - 0.243)	-	-	-
(ref: Junior high school)						
Senior high school	-	-	-	-0.150**	-0.150**	-0.149**
				(-0.299 - -	(-0.299 - -	(-0.298 - -
	-	-	-	0.000607)	0.000781)	0.000524)
College	-	-	-	-0.243*	-0.243*	-0.243*
	-	-	-	(-0.513 - 0.0270)	(-0.514 - 0.0271)	(-0.514 - 0.0271)
University	-	-	-	-0.488***	-0.489***	-0.487***
	-	-	-	(-0.731 - -0.245)	(-0.732 - -0.245)	(-0.730 - -0.244)
Marriage status						
(ref: unmarried/cohabitation)						
Currently married	-0.727*	-0.726*	-0.737*	-0.162	-0.162	-0.162
	(-1.567 - 0.113)	(-1.562 - 0.109)	(-1.571 - 0.0965)	(-0.468 - 0.143)	(-0.468 - 0.144)	(-0.468 - 0.144)
Divorce/Widow	0.713	0.719	0.710	0.613**	0.614**	0.618**

	(-0.275 - 1.702)	(-0.264 - 1.702)	(-0.272 - 1.691)	(0.0779 - 1.148)	(0.0783 - 1.149)	(0.0811 - 1.154)
Number of children below 16 in the household						
(ref: 0)						
1	0.0230	0.0215	0.0181	0.100	0.100	0.0994
	(-0.193 - 0.239)	(-0.193 - 0.236)	(-0.193 - 0.229)	(-0.0729 - 0.273)	(-0.0729 - 0.273)	(-0.0730 - 0.272)
2	-0.182	-0.180	-0.180	0.0154	0.0151	0.0156
	(-0.656 - 0.293)	(-0.655 - 0.295)	(-0.652 - 0.293)	(-0.183 - 0.214)	(-0.183 - 0.213)	(-0.183 - 0.215)
⩾3	-0.253	-0.253	-0.253	0.0709	0.0709	0.0707
	(-0.759 - 0.253)	(-0.757 - 0.252)	(-0.750 - 0.243)	(-0.137 - 0.278)	(-0.136 - 0.278)	(-0.136 - 0.278)
Not in full-time school	-	-	-	0.721	0.722	0.726
	-	-	-	(-0.274 - 1.715)	(-0.274 - 1.717)	(-0.272 - 1.724)
Employment status						
(ref: unemployment)						
In work	0.471	0.467	0.487	-0.784***	-0.785***	-0.783***
	(-0.628 - 1.569)	(-0.628 - 1.561)	(-0.598 - 1.572)	(-1.215 - -0.353)	(-1.217 - -0.354)	(-1.214 - -0.351)
Withdrawal from the labor market	0.586	0.578	0.588	-0.813***	-0.815***	-0.811***
	(-0.668 - 1.839)	(-0.671 - 1.827)	(-0.652 - 1.829)	(-1.351 - -0.274)	(-1.354 - -0.275)	(-1.350 - -0.273)
Non-entry into labor markets	-	-	-	-1.151**	-1.150**	-1.144**
	-	-	-	(-2.175 - -0.127)	(-2.175 - -0.125)	(-2.172 - -0.116)
Rural Hukou	0.0714	0.0717	0.0697	0.0547	0.0561	0.0551
	(-0.292 - 0.435)	(-0.291 - 0.434)	(-0.297 - 0.436)	(-0.189 - 0.298)	(-0.187 - 0.299)	(-0.188 - 0.299)
Having chronic conditions	1.295***	1.287***	1.282***	1.128***	1.126***	1.124***
	(0.887 - 1.702)	(0.877 - 1.697)	(0.877 - 1.687)	(0.780 - 1.476)	(0.778 - 1.474)	(0.776 - 1.473)
Constant	5.865***	5.886***	5.786***	2.551***	2.544***	2.474***
	(3.145 - 8.585)	(3.158 - 8.614)	(2.893 - 8.679)	(1.212 - 3.890)	(1.204 - 3.884)	(1.145 - 3.804)
Province FE	Yes	Yes	Yes	Yes	Yes	Yes
Month FE	Yes	Yes	Yes	Yes	Yes	Yes
Observations	3,985	3,985	3,985	9,670	9,670	9,670
R-squared	0.254	0.254	0.255	0.238	0.238	0.238

Note: OLS = Ordinary Least Squares. Coef. = Coefficient. 95% confidence intervals are reported in parentheses. *<0.1, **<0.05, ***<0.01. Local incidence rate of COVID-19 was constructed by taking the number of emerging local cases per 100,000 people in respondent's resident province within the specific window. Standard errors were clustered at the province level.

Table A4. Associations of COVID-19 Incidence Rate and Depression with OLS Model, by pre-pandemic depressive symptoms (China Family Panel Study, China. 2018 & 2020)

	(1)	(2)	(3)	(4)	(5)	(6)
Model	OLS (Coef.)					
Dependent variable	*Score of CES-D 2020*					
Subsample	Being Less Depressed in 2018 (CESD<8)			Being More depressed in 2018 (CESD≥8)		
7-day local incidence rate of COVID-19	1.722***			6.223***		
	(1.240 - 2.204)			(4.899 - 7.548)		
14-day local incidence rate of COVID-19		1.459***			3.306***	
		(0.728 - 2.189)			(1.890 - 4.722)	
28-day local incidence rate of COVID-19			0.597***			1.368
			(0.214 - 0.980)			(-0.883 - 3.618)
7-day imported incidence rate of COVID-19	1.051			0.371		
	(-0.757 - 2.860)			(-3.976 - 4.719)		
14-day imported incidence rate of COVID-19		0.484			-2.001	
		(-0.472 - 1.441)			(-6.805 - 2.803)	
28-day imported incidence rate of COVID-19			-0.0666			-1.883
			(-0.641 - 0.508)			(-6.518 - 2.753)
CES-D scores 2018	0.413***	0.413***	0.413***	0.441***	0.441***	0.441***
	(0.385 - 0.441)	(0.385 - 0.441)	(0.385 - 0.441)	(0.384 - 0.498)	(0.383 - 0.498)	(0.383 - 0.498)
Male	-0.270***	-0.270***	-0.270***	0.155	0.154	0.149
	(-0.421 - -0.119)	(-0.420 - -0.119)	(-0.420 - -0.119)	(-0.126 - 0.436)	(-0.128 - 0.437)	(-0.134 - 0.432)
Age	-0.0350	-0.0352	-0.0350	0.000729	-0.000721	-0.00165
	(-0.105 - 0.0353)	(-0.106 - 0.0352)	(-0.105 - 0.0352)	(-0.103 - 0.105)	(-0.105 - 0.103)	(-0.106 - 0.103)
Age square	0.000176	0.000179	0.000178	-0.000100	-8.49e-05	-7.18e-05
	(-0.000653 - 0.00101)	(-0.000651 - 0.00101)	(-0.000651 - 0.00101)	(-0.00132 - 0.00112)	(-0.00131 - 0.00114)	(-0.00130 - 0.00115)
Education attainment						
(ref: no school)						
Primary school	-0.396***	-0.397***	-0.396***	0.558**	0.561**	0.565**
	(-0.661 - -0.131)	(-0.661 - -0.133)	(-0.660 - -0.132)	(0.123 - 0.992)	(0.126 - 0.996)	(0.131 - 0.999)
Junior high school	-0.772***	-0.772***	-0.771***	0.00885	0.00349	0.00772
	(-1.098 - -0.447)	(-1.098 - -0.447)	(-1.097 - -0.446)	(-0.381 - 0.399)	(-0.386 - 0.393)	(-0.383 - 0.398)
Senior high school	-0.945***	-0.946***	-0.945***	-0.137	-0.142	-0.141
	(-1.275 - -0.616)	(-1.276 - -0.616)	(-1.275 - -0.616)	(-0.651 - 0.377)	(-0.656 - 0.372)	(-0.657 - 0.374)
College	-1.103***	-1.104***	-1.103***	-0.0341	-0.0423	-0.0439
	(-1.496 - -0.711)	(-1.498 - -0.710)	(-1.495 - -0.710)	(-0.727 - 0.659)	(-0.733 - 0.649)	(-0.730 - 0.642)
University	-1.290***	-1.291***	-1.289***	-0.397	-0.404	-0.401
	(-1.633 - -0.947)	(-1.635 - -0.946)	(-1.633 - -0.945)	(-1.100 - 0.306)	(-1.106 - 0.298)	(-1.101 - 0.300)
Marriage status						
(ref: unmarried/cohabitation)						
Currently married	-0.284	-0.283	-0.284	0.0261	0.0303	0.0286
	(-0.650 - 0.0828)	(-0.650 - 0.0839)	(-0.651 - 0.0832)	(-0.526 - 0.579)	(-0.522 - 0.583)	(-0.524 - 0.581)
Divorce/Widow	0.806***	0.807***	0.808***	0.867**	0.881**	0.883**

	(0.352 - 1.260)	(0.353 - 1.261)	(0.355 - 1.260)	(0.115 - 1.618)	(0.127 - 1.636)	(0.128 - 1.638)
Number of children below 16 in the household						
(ref: 0)						
1	0.167*	0.168*	0.166*	-0.110	-0.114	-0.112
	(-0.0194 - 0.354)	(-0.0200 - 0.356)	(-0.0205 - 0.352)	(-0.501 - 0.281)	(-0.501 - 0.274)	(-0.499 - 0.276)
2	0.180	0.180	0.180	-0.548**	-0.551**	-0.547**
	(-0.0845 - 0.444)	(-0.0852 - 0.446)	(-0.0852 - 0.445)	(-0.986 - -0.110)	(-0.985 - -0.116)	(-0.983 - -0.112)
⩾3	0.179	0.179	0.178	-0.478*	-0.482*	-0.479*
	(-0.114 - 0.473)	(-0.115 - 0.474)	(-0.115 - 0.471)	(-0.966 - 0.00965)	(-0.970 - 0.00588)	(-0.967 - 0.00887)
Not in full-time school	1.618***	1.617***	1.608***	-0.975	-0.977	-0.980
	(0.560 - 2.677)	(0.554 - 2.680)	(0.530 - 2.687)	(-3.232 - 1.282)	(-3.234 - 1.281)	(-3.235 - 1.276)
Employment status						
(ref: unemployment)						
In work	-0.312	-0.315	-0.311	-1.482***	-1.471***	-1.478***
	(-0.848 - 0.224)	(-0.854 - 0.224)	(-0.848 - 0.225)	(-2.462 - -0.502)	(-2.447 - -0.494)	(-2.456 - -0.499)
Withdrawal from the labor market	-0.350	-0.353	-0.349	-1.292***	-1.285***	-1.295***
	(-1.022 - 0.322)	(-1.029 - 0.323)	(-1.022 - 0.323)	(-2.225 - -0.359)	(-2.214 - -0.357)	(-2.226 - -0.363)
Non-entry into labor markets	0.0146	0.0132	0.00756	-3.361***	-3.362***	-3.375***
	(-1.153 - 1.182)	(-1.157 - 1.183)	(-1.175 - 1.190)	(-5.518 - -1.204)	(-5.516 - -1.208)	(-5.526 - -1.224)
Rural Hukou	0.0354	0.0364	0.0361	0.180	0.178	0.173
	(-0.209 - 0.279)	(-0.208 - 0.281)	(-0.209 - 0.281)	(-0.104 - 0.465)	(-0.103 - 0.459)	(-0.120 - 0.466)
Having chronic conditions	1.055***	1.053***	1.054***	1.388***	1.385***	1.383***
	(0.738 - 1.373)	(0.735 - 1.371)	(0.736 - 1.371)	(1.009 - 1.767)	(1.005 - 1.765)	(1.000 - 1.765)
Constant	3.135***	3.135***	3.082***	4.016**	4.045**	3.889**
	(1.396 - 4.874)	(1.390 - 4.879)	(1.332 - 4.833)	(0.891 - 7.140)	(0.915 - 7.175)	(0.714 - 7.064)
Province FE	Yes	Yes	Yes	Yes	Yes	Yes
Month FE	Yes	Yes	Yes	Yes	Yes	Yes
Observations	10,028	10,028	10,028	3,627	3,627	3,627
R-squared	0.118	0.118	0.118	0.148	0.148	0.148

Note: OLS = Ordinary Least Squares. Coef. = Coefficient. 95% confidence intervals are reported in parentheses. *<0.1, **<0.05, ***<0.01. Local incidence rate of COVID-19 was constructed by taking the number of emerging local cases per 100,000 people in respondent's resident province within the specific window. Standard errors were clustered at the province level.

Table A5. Associations of COVID-19 Incidence Rate and Depression with OLS Model, by chronic conditions (China Family Panel Study, China. 2018 & 2020)

	(1)	(2)	(3)	(4)	(5)	(6)
Model	OLS (Coef.)					
Dependent variable	*Score of CES-D 2020*					
Subsample	Having been diagnosed			Having not been diagnosed		
7-day local incidence rate of COVID-19	6.942***			2.160***		
	(4.014 - 9.869)			(1.677 - 2.643)		
14-day local incidence rate of COVID-19		-0.0798			2.036***	
		(-1.118 - 0.958)			(1.383 - 2.689)	
28-day local incidence rate of COVID-19			-0.112			0.909***
			(-0.766 - 0.541)			(0.341 - 1.476)
7-day imported incidence rate of COVID-19	-1.128			1.157		
	(-8.004 - 5.749)			(-0.596 - 2.910)		
14-day imported incidence rate of COVID-19		0.596			-0.0124	
		(-1.366 - 2.558)			(-1.284 - 1.259)	
28-day imported incidence rate of COVID-19			0.804			-0.600
			(-0.581 - 2.189)			(-1.717 - 0.516)
CES-D scores 2018	0.507***	0.508***	0.508***	0.451***	0.451***	0.450***
	(0.456 - 0.558)	(0.457 - 0.559)	(0.457 - 0.559)	(0.418 - 0.483)	(0.418 - 0.483)	(0.418 - 0.483)
Male	-0.342	-0.345	-0.345	-0.150**	-0.150**	-0.151**
	(-0.775 - 0.0909)	(-0.778 - 0.0875)	(-0.778 - 0.0884)	(-0.278 - - 0.0208)	(-0.279 - - 0.0210)	(-0.280 - - 0.0212)
Age	0.158	0.156	0.157	-0.0400	-0.0402	-0.0402
	(-0.0395 - 0.355)	(-0.0391 - 0.351)	(-0.0390 - 0.352)	(-0.0920 - 0.0120)	(-0.0922 - 0.0118)	(-0.0921 - 0.0116)
Age square	-0.00183	-0.00182	-0.00183	0.000276	0.000278	0.000280
	(-0.00408 - 0.000409)	(-0.00404 - 0.000404)	(-0.00406 - 0.000404)	(-0.000357 - 0.000908)	(-0.000355 - 0.000910)	(-0.000352 - 0.000912)
Education attainment						
(ref: no school)						
Primary school	0.0164	0.0211	0.0192	-0.0416	-0.0415	-0.0403
	(-0.523 - 0.556)	(-0.515 - 0.558)	(-0.518 - 0.556)	(-0.354 - 0.270)	(-0.353 - 0.270)	(-0.353 - 0.272)
Junior high school	-0.554	-0.555	-0.557	-0.475***	-0.475***	-0.474***
	(-1.298 - 0.189)	(-1.298 - 0.188)	(-1.300 - 0.186)	(-0.730 - -0.221)	(-0.729 - -0.221)	(-0.728 - -0.219)
Senior high school	-0.260	-0.258	-0.261	-0.687***	-0.688***	-0.688***
	(-1.009 - 0.488)	(-1.005 - 0.489)	(-1.008 - 0.487)	(-0.944 - -0.430)	(-0.946 - -0.431)	(-0.945 - -0.431)
College	-0.605	-0.630	-0.630	-0.744***	-0.745***	-0.743***
	(-1.639 - 0.428)	(-1.663 - 0.404)	(-1.664 - 0.403)	(-1.094 - -0.393)	(-1.096 - -0.394)	(-1.093 - -0.393)
University	-0.401	-0.411	-0.410	-1.026***	-1.028***	-1.027***
	(-1.481 - 0.678)	(-1.488 - 0.666)	(-1.485 - 0.666)	(-1.340 - -0.713)	(-1.343 - -0.714)	(-1.340 - -0.713)
Marriage status						
(ref: unmarried/cohabitation)						
Currently married	-0.968*	-0.959*	-0.958*	-0.180	-0.179	-0.180

	(-1.963 - 0.0275)	(-1.958 - 0.0398)	(-1.955 - 0.0383)	(-0.483 - 0.124)	(-0.482 - 0.124)	(-0.483 - 0.123)
Divorce/Widow	-0.147	-0.133	-0.133	0.883***	0.883***	0.885***
	(-1.999 - 1.704)	(-1.983 - 1.717)	(-1.982 - 1.717)	(0.389 - 1.377)	(0.389 - 1.378)	(0.390 - 1.379)
Number of children below 16 in the household						
(ref: 0)						
1	-0.0785	-0.0812	-0.0810	0.119	0.119	0.117
	(-0.647 - 0.490)	(-0.644 - 0.482)	(-0.644 - 0.482)	(-0.0321 - 0.269)	(-0.0328 - 0.271)	(-0.0328 - 0.267)
2	-0.0776	-0.0755	-0.0763	0.00513	0.00506	0.00519
	(-0.633 - 0.478)	(-0.631 - 0.480)	(-0.632 - 0.479)	(-0.255 - 0.265)	(-0.256 - 0.266)	(-0.256 - 0.266)
⩾3	-0.372	-0.372	-0.371	0.0657	0.0652	0.0642
	(-1.165 - 0.420)	(-1.161 - 0.416)	(-1.162 - 0.419)	(-0.126 - 0.258)	(-0.127 - 0.258)	(-0.127 - 0.256)
Not in full-time school	-1.003	-0.994	-0.996	0.969*	0.964*	0.944*
	(-4.260 - 2.253)	(-4.239 - 2.252)	(-4.240 - 2.247)	(-0.0455 - 1.984)	(-0.0550 - 1.983)	(-0.0877 - 1.976)
Employment status						
(ref: unemployment)						
In work	-1.258	-1.242	-1.244	-0.534**	-0.534**	-0.532**
	(-3.507 - 0.990)	(-3.467 - 0.984)	(-3.470 - 0.982)	(-1.014 - - 0.0540)	(-1.013 - - 0.0548)	(-1.011 - - 0.0532)
Withdrawal from the labor market	-1.358	-1.343	-1.345	-0.502*	-0.503*	-0.502*
	(-3.803 - 1.086)	(-3.764 - 1.078)	(-3.769 - 1.079)	(-1.087 - 0.0838)	(-1.089 - 0.0824)	(-1.087 - 0.0836)
Non-entry into labor markets	-1.756	-1.758	-1.753	-0.820	-0.824	-0.843
	(-5.792 - 2.279)	(-5.784 - 2.269)	(-5.779 - 2.273)	(-1.918 - 0.278)	(-1.926 - 0.277)	(-1.958 - 0.272)
Rural Hukou	0.243	0.223	0.222	0.0635	0.0649	0.0637
	(-0.145 - 0.631)	(-0.154 - 0.600)	(-0.153 - 0.597)	(-0.170 - 0.297)	(-0.167 - 0.297)	(-0.170 - 0.298)
Constant	3.007	3.166	3.173	3.336***	3.341***	3.270***
	(-1.838 - 7.852)	(-1.647 - 7.979)	(-1.631 - 7.976)	(1.804 - 4.868)	(1.804 - 4.878)	(1.729 - 4.810)
Province FE	Yes	Yes	Yes	Yes	Yes	Yes
Month FE	Yes	Yes	Yes	Yes	Yes	Yes
Observations	1,584	1,584	1,584	12,071	12,071	12,071
R-squared	0.305	0.304	0.304	0.227	0.227	0.227

Note: OLS = Ordinary Least Squares. Coef. = Coefficient. 95% confidence intervals are reported in parentheses. *<0.1, **<0.05, ***<0.01. Local incidence rate of COVID-19 was constructed by taking the number of emerging local cases per 100,000 people in respondent's resident province within the specific window. Standard errors were clustered at the province level.

Table A6. Using The Difference in CES-D Score as the Dependent Variable (China Family Panel Study, China. 2018 & 2020)

	(1)	(2)	(3)
	(CES-D 2020 - CES-D 2018) as Dependent Variable		
	Coef. (95% CI)	Coef. (95% CI)	Coef. (95% CI)
7-day Local Incidence Rate of COVID-19	3.796 (3.284~4.309)		
14-day Local Incidence Rate of COVID-19		2.841 (2.239~3.443)	
28-day Local Incidence Rate of COVID-19			1.028 (-0.383~2.440)

Note: OLS = Ordinary Least Squares. Coef. = Coefficient. 95% CI = 95% Confidence Interval. 95% CI are reported in parentheses. Local incidence rate of COVID-19 was constructed by taking the number of emerging local cases per 100,000 population in respondent's resident province within the specific window. Models controlled for: the CES-D score in 2018 wave, age, the square of age, gender, education attainment, marriage status, number of children under 16 years old in the household, whether registering into the full-time school, employment status, hukou status in 2018, whether having chronic disease diagnosed by doctors, the incidence rate of imported cases in the same window period, the province fixed effects, and the month fixed effects. Standard errors were clustered at the province level.

Table A7. Characteristics Mean Difference between 2020 Respondents vs. Nonrespondents (China Family Panel Study, China. 2018 & 2020)

	Whether Responding in 2020		**Difference**	**Adjusted Difference**
	**No**	**Yes**		
Age	38.04	38.46	0.421*	0.217
	[0.39]	[0.28]	[0.233]	[0.220]
Male	0.50	0.49	-0.005	-0.003
	[0.01]	[0.01]	[0.009]	[0.009]
Years of Education	8.86	9.41	0.551***	0.555***
	[0.35]	[0.28]	[0.151]	[0.103]
Agricultural Hukou	0.75	0.76	0.007	0.001
	[0.03]	[0.03]	[0.013]	[0.008]
Mean Score of CESD	5.50	5.49	-0.014	-0.037
	[0.17]	[0.15]	[0.079]	[0.061]
Having Chronic Conditions	0.11	0.12	0.009	0.007
	[0.01]	[0.01]	[0.006]	[0.005]

Note: SE = Standard Error. SEs are reported in brackets. * p<0.1, ** p<0.05, *** p<0.01. Adjusted difference shows the difference in mean after controlling for province fixed effects and clustering the standard error at the province level. People aged 16 to 58 in 2018 are included in this table.

Table A8. Robustness: The Subsample of Respondents Who Were both Tele-interviewed (China Family Panel Study, China. 2018 & 2020)

	(1)	(2)	(3)
Panel A: Score of CESD 2020 as Dependent Variable			
	Coef. (95% CI)	Coef. (95% CI)	Coef. (95% CI)
7-day Local Incidence Rate of COVID-19	3.233 (2.369~4.096)		
14-day Local Incidence Rate of COVID-19		3.690 (2.880~4.501)	
28-day Local Incidence Rate of COVID-19			1.768 (0.258~3.279)
Panel B: Indicator of Being More Depressed in 2020 (CES-D>=8) as Dependent Variable			
	AOR (95% CI)	AOR (95% CI)	AOR (95% CI)
7-day Local Incidence Rate of COVID-19	4.964 (2.172~11.349)		
14-day Local Incidence Rate of COVID-19		20.407 (10.272~40.538)	
28-day Local Incidence Rate of COVID-19			8.518 (4.086~17.757)

Note: OLS = Ordinary Least Squares. Coef. = Coefficient. AOR = Adjusted Odds Ratio. 95% CI = 95% Confidence Interval. 95% CI was reported in parentheses. Local incidence rate of COVID-19 was constructed by taking the number of emerging local cases per 100,000 population in respondent's resident province within the specific window. Models controlled for: the CES-D score in 2018 wave, age, the square of age, gender, education attainment, marriage status, number of children under 16 years old in the household, whether registering into the full-time school, employment status, hukou status in 2018, whether having chronic disease diagnosed by doctors, the incidence rate of imported cases in the same window period, the province fixed effect, and the month fixed effect. Standard errors were clustered at the province level. This table repeated the baseline with the respondents who were both tele-interviewed in 2018 and 2020, and the sample size is 3450.

Table A9. Associations of COVID-19 Incidence Rate and Depression with OLS Model and Logit Model (using a weighted subsample which has better national representativeness) (China Family Panel Study, China. 2018 & 2020)

	(1)	(2)	(3)	(4)	(5)	(6)
Model		OLS (Coef.)			Logit (AOR)	
Dependent variables		*Score of CES-D 2020*			*Indicator of Being More Depressed (CES-D>=8)*	
7-day local incidence rate of COVID-19	1.471**			6.983***		
	(0.249 - 2.693)			(1.638 - 29.77)		
14-day local incidence rate of COVID-19		4.005**			21.72**	
		(0.562 - 7.447)			(1.019 - 462.9)	
28-day local incidence rate of COVID-19			2.130			3.098
			(-0.445 - 4.705)			(0.560 - 17.15)
7-day imported incidence rate of COVID-19	1.387			3.657		
	(-4.844 - 7.618)			(0.166 - 80.30)		
14-day imported incidence rate of COVID-19		-0.289			1.918	
		(-3.438 - 2.860)			(0.386 - 9.528)	
28-day imported incidence rate of COVID-19			-1.177			1.644
			(-3.584 - 1.230)			(0.216 - 12.52)
CES-D scores 2018	0.472***	0.472***	0.472***	1.289***	1.289***	1.289***
	(0.436 - 0.509)	(0.436 - 0.509)	(0.435 - 0.509)	(1.258 - 1.320)	(1.258 - 1.321)	(1.258 - 1.320)
Male	-0.183**	-0.182**	-0.183**	0.890**	0.890**	0.890**
	(-0.360 - -0.00600)	(-0.359 - -0.00525)	(-0.359 - -0.00621)	(0.810 - 0.977)	(0.811 - 0.977)	(0.810 - 0.977)
Age	-0.0204	-0.0203	-0.0209	0.984	0.984	0.983
	(-0.0805 - 0.0397)	(-0.0803 - 0.0397)	(-0.0806 - 0.0389)	(0.942 - 1.027)	(0.942 - 1.027)	(0.942 - 1.027)
Age square	0.000118	0.000116	0.000123	1.000	1.000	1.000
	(-0.000595 - 0.000830)	(-0.000594 - 0.000827)	(-0.000585 - 0.000831)	(1.000 - 1.001)	(1.000 - 1.001)	(1.000 - 1.001)
Education attainment						
(ref: no school)						
Primary school	0.0136	0.0122	0.0137	1.072	1.070	1.071
	(-0.475 - 0.502)	(-0.476 - 0.500)	(-0.475 - 0.503)	(0.832 - 1.381)	(0.831 - 1.379)	(0.831 - 1.381)
Junior high school	-0.396**	-0.396**	-0.394**	0.896	0.896	0.896
	(-0.789 - -0.00316)	(-0.789 - -0.00358)	(-0.787 - -0.00139)	(0.736 - 1.089)	(0.736 - 1.089)	(0.736 - 1.090)
Senior high school	-0.539**	-0.542**	-0.540**	0.755**	0.754**	0.755**
	(-0.947 - -0.130)	(-0.950 - -0.133)	(-0.948 - -0.132)	(0.597 - 0.953)	(0.597 - 0.952)	(0.597 - 0.953)
College	-0.605***	-0.606***	-0.602***	0.779**	0.778**	0.779**
	(-1.029 - -0.182)	(-1.030 - -0.183)	(-1.024 - -0.180)	(0.618 - 0.981)	(0.616 - 0.981)	(0.618 - 0.981)
University	-0.863***	-0.863***	-0.857***	0.557***	0.557***	0.559***
	(-1.274 - -0.451)	(-1.274 - -0.451)	(-1.269 - -0.446)	(0.413 - 0.751)	(0.413 - 0.752)	(0.414 - 0.754)
Marriage status						
(ref: unmarried/cohabitation)						
Currently married	-0.262	-0.264	-0.262	0.775*	0.774*	0.775*
	(-0.591 - 0.0681)	(-0.594 - 0.0664)	(-0.591 - 0.0679)	(0.588 - 1.022)	(0.587 - 1.020)	(0.587 - 1.022)
Divorce/Widow	0.660**	0.656**	0.662**	1.356	1.353	1.359

	(0.155 - 1.164)	(0.150 - 1.162)	(0.157 - 1.167)	(0.918 - 2.003)	(0.915 - 2.001)	(0.921 - 2.006)
Number of children below 16 in the household						
(ref: 0)						
1	0.0477	0.0477	0.0478	1.057	1.056	1.056
	(-0.115 - 0.210)	(-0.114 - 0.210)	(-0.114 - 0.210)	(0.943 - 1.184)	(0.944 - 1.183)	(0.943 - 1.182)
2	-0.0784	-0.0773	-0.0777	1.007	1.007	1.007
	(-0.316 - 0.159)	(-0.315 - 0.160)	(-0.316 - 0.160)	(0.872 - 1.162)	(0.873 - 1.162)	(0.872 - 1.163)
⩾3	0.0791	0.0793	0.0769	1.127*	1.128*	1.127*
	(-0.131 - 0.289)	(-0.131 - 0.289)	(-0.132 - 0.286)	(0.997 - 1.274)	(0.998 - 1.275)	(0.997 - 1.274)
Not in full-time school	1.278*	1.268*	1.246*	2.244	2.248	2.254
	(-0.108 - 2.664)	(-0.109 - 2.646)	(-0.120 - 2.612)	(0.762 - 6.604)	(0.763 - 6.620)	(0.764 - 6.650)
Employment status						
(ref: unemployment)						
In work	-0.579**	-0.581**	-0.577**	0.700**	0.700**	0.701**
	(-1.135 - -0.0228)	(-1.134 - -0.0269)	(-1.131 - -0.0233)	(0.506 - 0.969)	(0.506 - 0.969)	(0.507 - 0.970)
Withdrawal from the labor market	-0.586*	-0.589*	-0.585*	0.663**	0.662**	0.663**
	(-1.205 - 0.0333)	(-1.205 - 0.0280)	(-1.201 - 0.0316)	(0.483 - 0.910)	(0.482 - 0.910)	(0.483 - 0.910)
Non-entry into labor markets	-0.329	-0.339	-0.367	0.755	0.756	0.754
	(-1.668 - 1.010)	(-1.672 - 0.993)	(-1.692 - 0.958)	(0.221 - 2.575)	(0.221 - 2.582)	(0.220 - 2.591)
Rural Hukou	0.208*	0.209*	0.212*	1.078	1.078	1.079
	(-0.00628 - 0.422)	(-0.00518 - 0.423)	(-0.00185 - 0.426)	(0.933 - 1.245)	(0.933 - 1.245)	(0.935 - 1.246)
Having chronic conditions	1.070***	1.068***	1.067***	1.601***	1.599***	1.597***
	(0.752 - 1.388)	(0.751 - 1.386)	(0.749 - 1.385)	(1.335 - 1.919)	(1.334 - 1.916)	(1.332 - 1.914)
Constant	2.383**	2.352**	2.116**	0.0674***	0.0648***	0.0556***
	(0.470 - 4.296)	(0.445 - 4.258)	(0.190 - 4.042)	(0.0176 - 0.258)	(0.0170 - 0.246)	(0.0145 - 0.214)
Province FE	Yes	Yes	Yes	Yes	Yes	Yes
Month FE	Yes	Yes	Yes	Yes	Yes	Yes
Observations	9,101	9,101	9,101	9,101	9,101	9,101
R-squared	0.261	0.262	0.262	0.163	0.163	0.163

Note: OLS = Ordinary Least Squares. Coef. = Coefficient. AOR = Adjusted Odds Ratio. 95% confidence intervals are reported in parentheses. *<0.1, **<0.05, ***<0.01. Local incidence rate of COVID-19 was constructed by taking the number of emerging local cases per 100,000 people in respondent's resident province within the specific window. Standard errors were clustered at the province level.

Table A10. Associations of COVID-19 Incidence Rate and Depression with OLS Model and Logit Model (high-incidence vs. low-incidence vs. no-incidence) (China Family Panel Study, China. 2018 & 2020)

	(1)	(2)	(3)	(4)	(5)	(6)
Model		OLS (Coef.)			Logit (AOR)	
Dependent variables		*Score of CES-D 2020*			*Indicator of Being More Depressed in 2020 (CES-D>=8)*	
7-day local incidence rate of COVID-19						
(ref: no cases)						
Lower than median	-0.0831			0.908		
	(-0.313 - 0.147)			(0.754 - 1.093)		
higher than median	0.307***			1.285***		
	(0.211 - 0.403)			(1.209 - 1.365)		
14-day local incidence rate of COVID-19						
(ref: no cases)						
Lower than median		-0.0429			0.917**	
		(-0.374 - 0.289)			(0.847 - 0.993)	
higher than median		0.214***			1.289***	
		(0.0851 - 0.344)			(1.207 - 1.378)	
28-day local incidence rate of COVID-19						
(ref: no cases)						
Lower than median			-0.0331			0.878*
			(-0.369 - 0.303)			(0.759 - 1.016)
higher than median			0.234			1.193***
			(-0.125 - 0.594)			(1.047 - 1.360)
7-day imported incidence rate of COVID-19	0.880			5.885***		
	(-0.920 - 2.681)			(1.645 - 21.06)		
14-day imported incidence rate of COVID-19		-0.0516			2.075	
		(-1.238 - 1.135)			(0.864 - 4.984)	
28-day imported incidence rate of COVID-19			-0.477			0.989
			(-1.659 - 0.704)			(0.327 - 2.994)
CES-D scores 2018	0.461***	0.461***	0.461***	1.275***	1.275***	1.274***
	(0.431 - 0.491)	(0.431 - 0.491)	(0.431 - 0.491)	(1.248 - 1.302)	(1.248 - 1.302)	(1.248 - 1.302)
Male	-0.166**	-0.166**	-0.166**	0.898***	0.898***	0.898***
	(-0.299 - -0.0321)	(-0.299 - -0.0323)	(-0.300 - -0.0324)	(0.838 - 0.962)	(0.838 - 0.962)	(0.838 - 0.962)
Age	-0.0240	-0.0243	-0.0241	0.984	0.984	0.984
	(-0.0757 - 0.0278)	(-0.0761 - 0.0275)	(-0.0757 - 0.0276)	(0.949 - 1.020)	(0.949 - 1.019)	(0.949 - 1.020)
Age square	8.79e-05	9.21e-05	9.04e-05	1.000	1.000	1.000
	(-0.000532 - 0.000708)	(-0.000528 - 0.000713)	(-0.000529 - 0.000709)	(1.000 - 1.001)	(1.000 - 1.001)	(1.000 - 1.001)
Education attainment						
(ref: no school)						
Primary school	-0.0458	-0.0454	-0.0445	1.067	1.067	1.068
	(-0.326 - 0.235)	(-0.326 - 0.235)	(-0.325 - 0.236)	(0.910 - 1.251)	(0.910 - 1.251)	(0.911 - 1.254)
Junior high school	-0.500***	-0.500***	-0.500***	0.870*	0.870*	0.870*

	(-0.767 - -0.233)	(-0.767 - -0.233)	(-0.768 - -0.232)	(0.746 - 1.015)	(0.745 - 1.015)	(0.745 - 1.017)
Senior high school	-0.665***	-0.666***	-0.667***	0.727***	0.726***	0.725***
	(-0.948 - -0.381)	(-0.950 - -0.383)	(-0.949 - -0.384)	(0.612 - 0.863)	(0.611 - 0.862)	(0.611 - 0.861)
College	-0.758***	-0.759***	-0.758***	0.697***	0.698***	0.697***
	(-1.113 - -0.403)	(-1.114 - -0.404)	(-1.113 - -0.404)	(0.557 - 0.873)	(0.557 - 0.873)	(0.556 - 0.873)
University	-0.995***	-0.997***	-0.999***	0.566***	0.565***	0.564***
	(-1.311 - -0.680)	(-1.314 - -0.680)	(-1.315 - -0.682)	(0.449 - 0.714)	(0.448 - 0.713)	(0.447 - 0.713)
Marriage status						
(ref: unmarried/cohabitation)						
Currently married	-0.225	-0.223	-0.225	0.812	0.814	0.813
	(-0.536 - 0.0856)	(-0.533 - 0.0866)	(-0.535 - 0.0865)	(0.621 - 1.063)	(0.622 - 1.065)	(0.621 - 1.063)
Divorce/Widow	0.786***	0.788***	0.788***	1.403*	1.404*	1.404*
	(0.259 - 1.313)	(0.261 - 1.315)	(0.260 - 1.316)	(0.988 - 1.991)	(0.988 - 1.995)	(0.988 - 1.995)
Number of children below 16 in the household						
(ref: 0)						
1	0.0851	0.0852	0.0851	1.041	1.042	1.040
	(-0.0549 - 0.225)	(-0.0555 - 0.226)	(-0.0541 - 0.224)	(0.952 - 1.138)	(0.952 - 1.140)	(0.952 - 1.137)
2	-0.0177	-0.0173	-0.0173	0.995	0.995	0.994
	(-0.261 - 0.226)	(-0.262 - 0.227)	(-0.262 - 0.227)	(0.860 - 1.151)	(0.859 - 1.151)	(0.859 - 1.150)
⩾3	-0.000189	-0.000726	-0.000628	1.062	1.062	1.061
	(-0.220 - 0.220)	(-0.221 - 0.219)	(-0.221 - 0.219)	(0.950 - 1.188)	(0.949 - 1.189)	(0.949 - 1.187)
Not in full-time school	0.818	0.813	0.799	1.211	1.211	1.215
	(-0.191 - 1.827)	(-0.198 - 1.823)	(-0.227 - 1.824)	(0.538 - 2.729)	(0.537 - 2.733)	(0.533 - 2.769)
Employment status						
(ref: unemployment)						
In work	-0.607***	-0.607***	-0.605***	0.722**	0.721**	0.722**
	(-1.017 - -0.197)	(-1.016 - -0.198)	(-1.010 - -0.200)	(0.535 - 0.973)	(0.535 - 0.972)	(0.537 - 0.971)
Withdrawal from the labor market	-0.578**	-0.578**	-0.577**	0.705**	0.704**	0.705**
	(-1.082 - -0.0739)	(-1.082 - -0.0737)	(-1.076 - -0.0787)	(0.506 - 0.982)	(0.505 - 0.983)	(0.507 - 0.980)
Non-entry into labor markets	-0.985*	-0.986*	-0.997*	0.398*	0.398*	0.400*
	(-2.032 - 0.0622)	(-2.035 - 0.0624)	(-2.059 - 0.0652)	(0.145 - 1.094)	(0.145 - 1.096)	(0.144 - 1.112)
Rural Hukou	0.0759	0.0755	0.0764	1.005	1.005	1.006
	(-0.136 - 0.287)	(-0.136 - 0.287)	(-0.135 - 0.287)	(0.856 - 1.180)	(0.857 - 1.180)	(0.857 - 1.180)
Having chronic conditions	1.176***	1.175***	1.176***	1.719***	1.718***	1.720***
	(0.877 - 1.476)	(0.875 - 1.475)	(0.877 - 1.475)	(1.478 - 2.000)	(1.476 - 2.000)	(1.478 - 2.001)
Constant	3.264***	3.264***	3.096***	0.137***	0.139***	0.118***
	(1.810 - 4.719)	(1.776 - 4.751)	(1.549 - 4.644)	(0.0539 - 0.350)	(0.0538 - 0.358)	(0.0440 - 0.317)
Province FE	Yes	Yes	Yes	Yes	Yes	Yes
Month FE	Yes	Yes	Yes	Yes	Yes	Yes
Observations	13,655	13,655	13,655	13,655	13,655	13,655
R-squared	0.255	0.255	0.255	0.155	0.155	0.155

Note: OLS = Ordinary Least Squares. Coef. = Coefficient. AOR = Adjusted Odds Ratio. 95% confidence intervals are reported in parentheses. *<0.1, **<0.05, ***<0.01. Local incidence rate of COVID-19 was constructed by taking the number of emerging local cases per 100,000 people in respondent's resident province within the specific window. Standard errors were clustered at the province level.

Table A11. Associations of COVID-19 Incidence Rate and Depression with OLS Model and Logit Model (Random-Effects) (China Family Panel Study, China. 2018 & 2020)

Model	OLS (Coef.)			Logit (AOR)		
Dependent variables	*Score of CES-D 2020*			*Indicator of Being More Depressed in 2020 (CES-D>=8) 2020*		
7-day local incidence rate of COVID-19	2.729*			6.583***		
	(-0.0802 - 5.538)			(3.160 - 13.71)		
14-day local incidence rate of COVID-19		1.759*			5.548***	
		(-0.244 - 3.763)			(2.645 - 11.63)	
28-day local incidence rate of COVID-19			0.786			2.601**
			(-0.716 - 2.288)			(1.020 - 6.634)
7-day imported incidence rate of COVID-19	0.835			5.067***		
	(-1.050 - 2.720)			(1.774 - 14.47)		
14-day imported incidence rate of COVID-19		0.113			2.039*	
		(-1.007 - 1.232)			(0.980 - 4.243)	
28-day imported incidence rate of COVID-19			-0.0766			1.298
			(-0.947 - 0.794)			(0.552 - 3.051)
CES-D scores 2018	0.465***	0.465***	0.464***	1.275***	1.275***	1.275***
	(0.436 - 0.493)	(0.436 - 0.493)	(0.436 - 0.493)	(1.248 - 1.303)	(1.248 - 1.303)	(1.248 - 1.303)
Male	-0.159**	-0.159**	-0.160**	0.901***	0.901***	0.900***
	(-0.286 - -0.0327)	(-0.285 - -0.0326)	(-0.286 - -0.0333)	(0.841 - 0.964)	(0.842 - 0.964)	(0.841 - 0.964)
Age	-0.0196	-0.0198	-0.0199	0.985	0.984	0.984
	(-0.0679 - 0.0287)	(-0.0680 - 0.0285)	(-0.0681 - 0.0283)	(0.953 - 1.018)	(0.953 - 1.017)	(0.953 - 1.017)
Age square	3.27e-05	3.51e-05	3.78e-05	1.000	1.000	1.000
	(-0.000542 - 0.000607)	(-0.000539 - 0.000609)	(-0.000536 - 0.000611)	(1.000 - 1.001)	(1.000 - 1.001)	(1.000 - 1.001)
Education attainment						
(ref: no school)						
Primary school	-0.0646	-0.0655	-0.0636	1.057	1.056	1.057
	(-0.327 - 0.198)	(-0.328 - 0.197)	(-0.326 - 0.199)	(0.904 - 1.236)	(0.903 - 1.235)	(0.903 - 1.236)
Junior high school	-0.552***	-0.552***	-0.550***	0.855**	0.854**	0.854**
	(-0.808 - -0.295)	(-0.809 - -0.296)	(-0.806 - -0.295)	(0.734 - 0.995)	(0.733 - 0.994)	(0.733 - 0.996)
Senior high school	-0.699***	-0.700***	-0.700***	0.718***	0.717***	0.717***
	(-0.964 - -0.434)	(-0.966 - -0.435)	(-0.964 - -0.436)	(0.605 - 0.852)	(0.603 - 0.851)	(0.604 - 0.851)
College	-0.795***	-0.797***	-0.796***	0.688***	0.687***	0.687***
	(-1.123 - -0.467)	(-1.126 - -0.468)	(-1.123 - -0.468)	(0.549 - 0.862)	(0.547 - 0.863)	(0.547 - 0.862)
University	-1.040***	-1.041***	-1.039***	0.559***	0.558***	0.559***
	(-1.335 - -0.744)	(-1.338 - -0.745)	(-1.335 - -0.744)	(0.443 - 0.706)	(0.441 - 0.706)	(0.443 - 0.707)
Marriage status						
(ref: unmarried/cohabitation)						
Currently married	-0.264*	-0.262*	-0.261*	0.807	0.808	0.808
	(-0.568 - 0.0399)	(-0.566 - 0.0415)	(-0.565 - 0.0430)	(0.618 - 1.054)	(0.618 - 1.056)	(0.618 - 1.055)
Divorce/Widow	0.745***	0.748***	0.753***	1.391*	1.391*	1.395*
	(0.238 - 1.252)	(0.241 - 1.255)	(0.247 - 1.259)	(0.984 - 1.965)	(0.985 - 1.966)	(0.988 - 1.969)

Number of children below 16 in the household						
(ref: 0)						
1	0.0920	0.0919	0.0895	1.045	1.045	1.043
	(-0.0466 - 0.231)	(-0.0472 - 0.231)	(-0.0473 - 0.226)	(0.955 - 1.143)	(0.955 - 1.144)	(0.954 - 1.140)
2	-0.0144	-0.0145	-0.0178	0.997	0.997	0.995
	(-0.254 - 0.225)	(-0.256 - 0.227)	(-0.258 - 0.222)	(0.859 - 1.157)	(0.858 - 1.158)	(0.857 - 1.155)
⩾3	0.0419	0.0411	0.0353	1.073	1.073	1.070
	(-0.206 - 0.290)	(-0.209 - 0.291)	(-0.214 - 0.284)	(0.949 - 1.212)	(0.948 - 1.215)	(0.946 - 1.210)
Not in full-time school	0.830*	0.826*	0.820	1.215	1.214	1.214
	(-0.141 - 1.801)	(-0.148 - 1.799)	(-0.159 - 1.800)	(0.532 - 2.772)	(0.531 - 2.778)	(0.536 - 2.751)
Employment status						
(ref: unemployment)						
In work	-0.581***	-0.581***	-0.579***	0.725**	0.725**	0.726**
	(-0.969 - -0.192)	(-0.969 - -0.193)	(-0.965 - -0.192)	(0.540 - 0.975)	(0.538 - 0.976)	(0.540 - 0.977)
Withdrawal from the labor market	-0.572**	-0.573**	-0.569**	0.705**	0.704**	0.706**
	(-1.048 - -0.0959)	(-1.051 - -0.0955)	(-1.044 - -0.0952)	(0.507 - 0.980)	(0.505 - 0.983)	(0.507 - 0.983)
Non-entry into labor markets	-0.927*	-0.929*	-0.933*	0.405*	0.405*	0.406*
	(-1.923 - 0.0686)	(-1.927 - 0.0685)	(-1.937 - 0.0712)	(0.145 - 1.135)	(0.144 - 1.138)	(0.146 - 1.129)
Rural Hukou	0.0839	0.0852	0.0810	1.005	1.007	1.005
	(-0.109 - 0.277)	(-0.108 - 0.278)	(-0.114 - 0.276)	(0.862 - 1.173)	(0.864 - 1.173)	(0.863 - 1.172)
Having chronic conditions	1.176***	1.175***	1.173***	1.716***	1.715***	1.712***
	(0.891 - 1.461)	(0.889 - 1.460)	(0.888 - 1.458)	(1.473 - 2.000)	(1.471 - 2.000)	(1.466 - 1.998)
The second industry share in GDP	-0.0129	-0.0132	-0.0128	1.008	1.008	1.008
	(-0.0664 - 0.0405)	(-0.0671 - 0.0407)	(-0.0666 - 0.0411)	(0.978 - 1.038)	(0.977 - 1.039)	(0.978 - 1.039)
The third industry share in GDP	-0.0182	-0.0171	-0.0167	0.996	0.996	0.996
	(-0.0688 - 0.0325)	(-0.0680 - 0.0337)	(-0.0681 - 0.0346)	(0.968 - 1.025)	(0.967 - 1.026)	(0.965 - 1.028)
ln(GDP per capita)	-0.224	-0.212	-0.212	0.801	0.810	0.814
	(-0.777 - 0.329)	(-0.769 - 0.345)	(-0.767 - 0.343)	(0.572 - 1.122)	(0.576 - 1.139)	(0.582 - 1.140)
Population density	0.282	0.298	0.293	1.087	1.108	1.121
	(-0.153 - 0.717)	(-0.143 - 0.739)	(-0.163 - 0.749)	(0.865 - 1.366)	(0.879 - 1.396)	(0.900 - 1.397)
Constant	5.657**	5.365**	5.369**	1.225	0.929	0.793
	(1.321 - 9.994)	(1.000 - 9.730)	(1.060 - 9.679)	(0.0608 - 24.67)	(0.0482 - 17.91)	(0.0743 - 8.460)
Observations	13,655	13,655	13,655	13,655	13,655	13,655

Note: OLS = Ordinary Least Squares. Coef. = Coefficient. AOR = Adjusted Odds Ratio. 95% confidence intervals are reported in parentheses. *<0.1, **<0.05, ***<0.01. Local incidence rate of COVID-19 was constructed by taking the number of emerging local cases per 100,000 people in respondent's resident province within the specific window. Standard errors were clustered at the province level.
